# Gluteal compartment syndrome: a case report

**DOI:** 10.1186/1757-1626-2-190

**Published:** 2009-11-10

**Authors:** Nadia M Mustafa, Aerin Hyun, James S Kumar, Lalitha Yekkirala

**Affiliations:** 1 Internal Medicine Residency Program, College of Medicine, University of Illinois at Urbana-Champaign, 611 W Park St, Urbana, IL 61801, USA; 2 Associate Professor, College of Medicine, University of Illinois at Urbana-Champaign, 611 W Park St, Urbana, IL 61801, USA

## Abstract

**Introduction:**

Gluteal compartment syndrome is a rare, often unrecognized syndrome that may manifest as renal failure, sepsis, and death. Delay in diagnosis can result in significant morbidity and possible mortality. We report a case of occult gluteal compartment syndrome causing unresolving rhabdomyolysis.

**Case Presentation:**

A 50-year-old Caucasian American man with history of chronic obstructive pulmonary disease was admitted status post fall and loss of consciousness for an unknown duration. Initial work-up revealed severe rhabdomyolysis, opioid abuse and acute renal failure. Inspite of three days of intensive therapy his condition did not improve and his renal failure worsened. On improvement of his condition three days later, he indicated some discomfort in his right hip. Physical examination was significant for swelling of the right gluteal region, which was tender and firm on palpation. A non-contrast CT scan showed evidence of gluteal compartment syndrome and emergent surgery resulted in significant improvement of his condition.

**Conclusion:**

Gluteal compartment syndrome most commonly occurs in individuals with altered mental status due to drugs or alcohol, who remain in one position for an extended period of time. This prolonged compression leads to muscle damage, edema, and a full-blown compartment syndrome. Due to its anatomic location and rarity, diagnosis is often missed or delayed, resulting in significant morbidity and possible mortality. The mainstay of treatment is fasciotomy.

## Introduction

Gluteal compartment syndrome as a result of fall or trauma has rarely been reported. It is mostly associated with drug overdose, alcohol intoxication and prolonged immobilization. It is imperative to identify the condition as early in the process as possible and to treat it with the appropriate interventions. Failure to do so may result in dangerous, irreversible sequelae such as rhabdomyolysis, renal failure, multiple-organ failure, and even possibly death.

## Case Presentation

A 50 year-old Caucasian American man with history of chronic obstructive pulmonary disease was admitted for loss of consciousness. He was found on the floor by his son, who brought him to the emergency department. The son denied noticing any tongue bite or bowel or bladder incontinence. The patient was unresponsive on arrival to the emergency department.

The patient's home medications included albuterol inhaler as needed. He was smoking two packs of cigarettes daily, but the son denied history of alcohol or illicit drug intake. His vitals on presentation were: BP - 90/50 mmHg, HR - 116/min, RR - 28/min and Temp - 98.8°F, and he was saturating 92 percent on 2L oxygen via nasal cannula.

His physical examination was significant for unresponsiveness, crackles in his left lung base and scattered wheezes throughout the lungs. His lab work showed a high WBC of 26,400/L, with 72.5% neutrophils and 13.5% bands, potassium of 5.3 mEq/L, BUN of 15 mg/dL and creatinine of 1.9 mg/dL (his baseline creatinine was 1.1 mg/dL). Urine toxicology screen was positive for opioid. Other labs included elevated creatine kinase of 15,860 U/L and elevated myoglobin of 46,651 ng/ml. Urine was positive for pneumococcal antigen. CT scan of the head without contrast was normal. Chest x-ray showed an infiltrate in the left mid lung field.

The patient was admitted with a diagnosis of chronic obstructive pulmonary disease exacerbation secondary to pneumonia, fall with loss of consciousness secondary to severe sepsis, opioid abuse and acute renal failure secondary to rhabdomyolysis. He was started on intravenous steroids, antibiotics and bronchodilators. He was given intravenous fluid for his rhabdomyolysis and hypotension. Despite aggressive treatment of rhabdomyolysis his creatine kinase increased from 15,000 U/L on the day of admission to 45,000 U/L on day two and 80,000 U/L on day three. His serum creatinine level increased from 1.9 mg/dL on admission to 2.3 mg/dL on day two and 2.6 mg/dL on day three.

**Figure 1:**
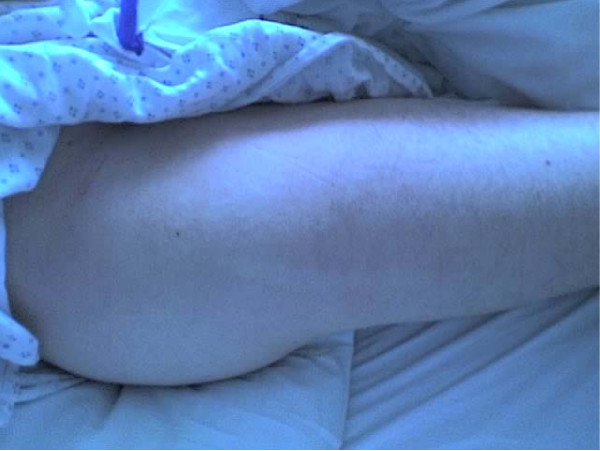
**Right hip of the patient showing increased swelling, tenderness and firmness on palpation**.

On day three, the patient became slightly responsive and started complaining of pain in his right hip. On inspection, the patient's right hip was swollen, and it was very firm and tender on palpation (Figure 1).

**Figure 2:**
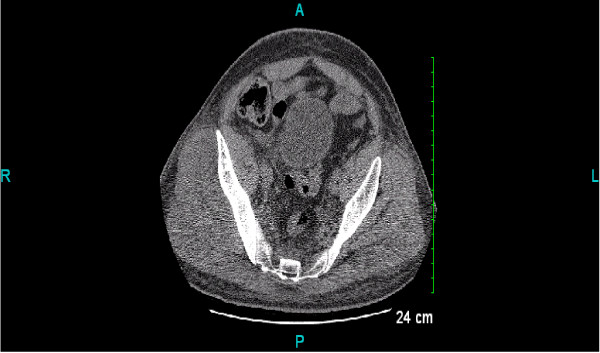
**CT scan of the pelvis - Fullness of the right gluteal maximus and medius muscles, possibly secondary to edema or inflammation or compartment syndrome**.

CT scan of pelvis was done which demonstrated fullness of the right gluteal muscles secondary to edema or inflammation or compartment syndrome (Figure 2).

**Figure 3:**
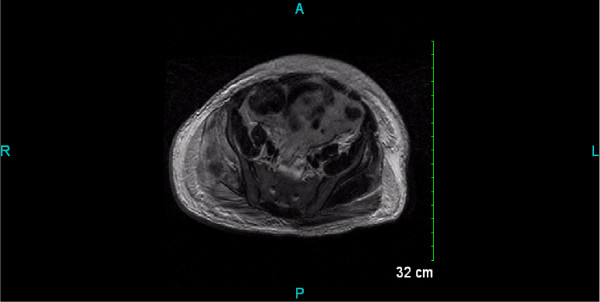
**MRI pelvis**. Enlargement of right gluteus minimus and medius muscles secondary to edema or hemorrhage with compartment syndrome.

An MRI of the pelvis was recommended for further evaluation which showed enlargement of right gluteus minimus and medius muscles secondary to edema or hemorrhage with compartment syndrome (Figure 3). Gluteal compartment pressure was not checked as there seemed to be enough evidence from physical examination and rising serum creatinine and creatine kinase levels, that the patient's worsening rhabdomyolysis was secondary to the gluteal compartment syndrome.

The patient was taken to the operating room where he underwent multiple fasciotomies. A large area of clot was noted between gluteus maximus and medius which was evacuated and the area was irrigated. The patient's creatine kinase and serum creatinine level improved to normal on discharge to home, five days after the surgical procedure. On follow-up visit to the office, two weeks after the patient's discharge, he was doing fine with normalization of his renal function and absence of gait abnormality, sensory dysfunction or muscle weakness.

## Discussion

Compartment syndrome results from increased interstitial fluid pressure of an osseofascial compartment, leading to compromise of the microcirculation and ultimately in tissue necrosis. The syndrome's occurrence in limb regions has been well documented, but has seldom been noted in the literature as occurring in the gluteal region [1]. To date, 44 cases have been reported in works published in English journals, five of which have been cases of bilateral gluteal compartment syndrome [1,2].

There exist three compartments in the gluteal region: the gluteus maximus, medius/minimus and tensor fascia lata [1]. The anatomic constraints of these compartments do not accommodate excessive swelling and edema, and when this occurs it causes increased compartment pressure, decreased blood flow, and finally ischemia [3]. Whiteside et al. have showed that a muscle can only survive 4 hours of temporary ischemia before minor functional and histological damage give way to a fatal ischemia. Neurons are more sensitive to hypoxia, and evidence of their sensory deficit may be observed in as soon as 33 min [1,4]. Gluteal compartment syndrome has most commonly been described in the literature as occurring after prolonged immobility associated with substance abuse, improper operative positioning, sickle cell-induced infarct, post-traumatic and spontaneous superior gluteal artery rupture, exercise, and post-arterial embolization of the internal iliac artery prior to abdominal aortic aneurysm repair [5]. Trauma is rarely associated with this syndrome.

Physical examination includes severe buttock pain at rest associated with painful movement of the hip, as well as bruising, paresthesia and tense swelling of the buttocks. With progressive increase in compartmental pressures, ischemic changes occur in the sciatic nerve, resulting in sciatic nerve palsy [5]. No major nerves or vessels exist in the gluteal compartment. However, neurologic findings have been reported in approximately 50 percent of reported cases. Since the sciatic nerve lies posterior to the gluteus maximus and not within the compartment, it should therefore not be susceptible to increased pressure and/or ischemic effects associated with gluteal compartment syndromes. The sciatic nerve does however lie between the gluteus maximus and the pelvis external rotator complex, thereby making it susceptible to compression by swelling of the gluteal muscles. This may result in a compression-induced neuropathy [6].

A large amount of intravascular fluid may be lost into the gluteal compartment, resulting in third-space fluid loss and hypotension. The infarcted muscle causes cellular breakdown with myoglobin and potassium release. The resulting hyperkalemia causes acidosis, and myoglobin deposits in the distal renal tubules, which may result in acute renal failure. Thus, a gluteal compartment syndrome may lead to significant morbidity and mortality in affected patients [3]. Blood pressure should be monitored in these patients, as well as observation for any evidence rhabdomyolysis in order to guard against progression to renal failure. Patients should be adequately hydrated, and renal dialysis is indicated in cases of advanced renal failure [6].

Compartment syndrome diagnosis has to date remained largely based on clinical findings [1,3]. Measurement of compartment pressures will aid in the diagnosis of compartment syndrome. Pressures of ≥ 30 mm Hg are considered suggestive of a compartment syndrome and necessitate fasciotomy. The measured compartmental pressures must also be correlated with the patient's blood pressure at the time of measurement [1,5,7]. The earlier in the ischemic process the fasciotomy occurs, the less the amount of irreversible damage done to the affected muscle(s) and systemic side effects. All three of the gluteal compartments (maximus, medius, and the minimus) must be decompressed by fasciotomy in order to treat compartment syndrome occurring in the buttock [3]. Prompt intervention becomes vital for tissue and functional salvage [6]. Appropriate intervention includes adequate exposure and decompression. Urgent angiography and embolization should be done in the setting of profuse bleeding. Ligation of the internal iliac artery has been proposed as a life-saving maneuver. Even post-fasciotomy, marginally viable tissues do not always immediately resolve, particularly in the presence of hypotension [1]. There currently exists a limited amount of documented proof supporting the use of hyperbaric oxygen during the initial phases of compartment syndromes. This approach to treatment may be considered as adjuvant therapy until further studies have been conducted regarding its efficacy in this context [1].

## Conclusion

In all cases of compartment syndrome, it becomes imperative to identify the condition as early in the process as possible and to treat it with the appropriate interventions. Failure to do so may result in dangerous, irreversible sequelae such as rhabdomyolysis, renal failure, multiple-organ failure, and even possibly death [5].

## Abbreviations

MRI: magnetic resonance imaging;

## Competing interests

The authors declare that they have no competing interests.

## Authors' contributions

NM - Took care of the patient while in the hospital, wrote the manuscript, collected all the relevant data, did literature review and finalized the manuscript for submission to the journal. AH - Involved in collecting data, do literature review, correcting any mistakes. JK and LY- Involved in giving intellectual advice and correcting any mistakes.

## Consent

Written informed consent was obtained from the patient for publication of this case report and accompanying images. A copy of the written consent is available for review by the editor-in-Chief of this journal.
